# A brief review of malaria epidemiological trend in Kermanshah province, Iran, 1986–2014

**DOI:** 10.25122/jml-2021-0374

**Published:** 2022-03

**Authors:** Naser Nazari, Yazdan Hamzavi, Mansour Rezaei, Peyvand Khoshbo

**Affiliations:** 1. Infectious Diseases Research Center, Kermanshah University of Medical Sciences, Kermanshah, Iran; 2. Parasitology and Mycology Department, School of Medicine, Kermanshah University of Medical Science, Kermanshah, Iran; 3. Social Development and Health Promotion Research Center, Kermanshah University of Medical Sciences, Kermanshah, Iran; 4. Students Research Committee, Kermanshah University of Medical Sciences, Kermanshah, Iran

**Keywords:** malaria, epidemiologic trend, plasmodium SP, Kermanshah

## Abstract

Malaria is the most severe protozoan disease in the world. As a result of strict malaria control programs, malaria's epidemiological model has changed. Knowing this epidemiological model and its effects will help us predict and prevent a new epidemic. This research was conducted to review the epidemiological trend of malaria in the Kermanshah province of Iran and some of its effective factors. Data were extracted from the registers in the disease control unit of the province, national population census, and annual rainfall report. The data was processed by SPSS16. There has been an evident decrease in malaria cases over the last 30 years in Kermanshah. This decreasing trend began especially after 1994, and since then, just 6% of all cases have happened. Between 1990 to 1997, an epidemic occurred, and more than 80% of positive cases were registered in these years. *P. vivax* malaria was the most common type (99.32%), and *P. falciparum* malaria was the second, with a very egregious difference (0.68%). The average age was 23.1 years, and men were infected twice more than women. There was a positive relation between annual rainfall level and positive malaria cases in these cities. After the major changes in malaria control programs in Kermanshah province, the number of positive cases fell noticeably, and now it is in the elimination stage. All cases of malaria were imported in the last years, and no resistance type was ever seen.

## INTRODUCTION

According to the last World Health Organization (WHO) report, there were 229 million malaria cases worldwide in 2019, indicating a slight decrease from 238 million in 2000 [1]. Nowadays, malaria is the most severe protozoan infection globally, and it affects many people worldwide every year [2]. However, between 2000 and 2015, malaria incidence among populations at risk fell by 37% globally; and mortality rates decreased by 60% and 65% in children younger than five years [3].

Countries in Sub-Saharan Africa have the highest burden of disease, with the Democratic Republic of the Congo and Nigeria accounting for 34% of the estimated global malaria deaths [1]. Decreases in case incidence and mortality rates were lowest in countries with the largest malaria cases and deaths in 2000. The incidence needs to be greatly decreased in these countries if global progress is to improve [4]. Countries that have eliminated malaria achieved remarkable success in reducing associated malaria burdens between 2000 and 2010. Malaria caused by *Plasmodium vivax* is increasingly imported from settings outside Sub-Saharan Africa and clustered in small geographical areas or subpopulations, often with shared social, behavioral, and geographical risk characteristics. The shift in the populations most at risk of malaria raises important questions for malaria-eliminating countries since traditional control interventions are less effective. The elimination approaches need to be aligned with these changes by developing and adopting novel strategies and methods. Knowing the changing epidemiological trends of malaria in the eliminating countries will improve the target of interventions to continue shrinking the malaria map [5]. Other challenges include high economic charges of a control plan and resistance to at least one of the insecticides in 60 of 78 studied countries. Artemisinin resistance is seen in some countries, and a high amount of treatment failure by artesunate is seen in eastern Asian countries. Resistance to high-dose Chloroquine is demonstrated in 10 countries [4]. To emphasize the remaining challenge and initiate the "Eradication" phase, the WHO has created a new strategy for malaria in 2016–2030 with bigger goals [4].

### Factors affecting the malaria epidemic

Malaria epidemics occur when climate and other conditions favor transmission in areas with little or no immunity to malaria [3]. Transmission is more intense in places where the mosquito lifespan is longer and prefers to bite humans rather than animals. The long lifespan and strong human-biting habit of the African vector species is the main reason for having nearly 90% of the world's malaria cases in Africa [3]. The transmission also depends on climatic conditions that may affect the lifespan and number of mosquitos. In many places, transmission is seasonal, with the peak during and just after the rainy season [3]. Human immunity is another important factor, especially among adults in areas of moderate or intense transmission conditions. Partial immunity can be developed after exposure, and although it does not provide complete protection, it reduces the risk of severe disease. Subsequently, most malaria deaths in Africa occur in young children, whereas all age groups are at risk in areas with less transmission and low immunity [3].

### Malaria transmission in the world

Between 2000 and 2015, malaria incidence among populations at risk fell by 37% globally, and mortality rates decreased by 60% and 65% in children under five years [3]. In 2015, approximately 3.2 billion people – nearly half of the world's population – were at risk of malaria. Most malaria cases and deaths occurred in Sub-Saharan Africa. However, Asia, Latin America, and to a lesser extent, the Middle East are also at risk. In 2015, 97 countries and territories had ongoing malaria transmission [3]. The number of positive malaria cases was 214 million, causing 438,000 deaths [4].

### Malaria transmission in Iran

In Iran, the Malaria Control Program started extensively in 1951. Before the program started, there was an infection rate of 4 million people per year, according to the estimations made in Iran. The areas involved were southern areas near the Persian Gulf, Azerbaijan, and coastal areas of the Caspian Sea. Subsequent malaria metric studies of the Institute of Malariology demonstrated that the disease has dispersed in many villages all over the country [6]. Following extensive malaria control programs, which was named "Malaria Eradication" in the first years, the disease was eliminated in most parts of the country. After 1972, the disease was under control in most parts of the country, and local transmission was limited to some endemic parts, mainly in Sistan and Baluchestan, Hormozgan, and Kerman Provinces [7]. Iran has reached the "elimination" phase with an over 75% decrease in malaria-positive cases between 2000 and 2015 [4]. In 2012, 1629 cases of malaria were reported in Iran, and it reached 519 cases by the year 2013 [8]. This decreasing trend continued, and in 2014 only 358 cases of malaria were reported [4].

Although the number of positive malaria cases has greatly decreased, we cannot control the spread of the disease. Therefore, in this research, we try to draw an epidemiological pattern for malaria in Kermanshah and provide a simple pattern used in official malaria elimination programs.

## MATERIAL AND METHODS

This is a descriptive-analytic study carried out during 30 years in Kermanshah province in the west of Iran. Kermanshah province has a hot and dry summer, where the mean rainfall is 50 mm. The altitude ranges 1200 meters above sea level. All information regarding malaria disease was collected from patients visiting the hygienic centers of Kermanshah province from 1986 to 2014, in different cities from Kermanshah province.

The data collection form included patient name, age, sex, location of the disease, type of *Plasmodium*, type of transmission, nationality, type of smear, date of infection etc, recorded in Kermanshah hygienic centers, Kermanshah province, Iran (1986–2014). The diagnosis of malaria infection was made after assessing clinical symptoms and was confirmed by the type of *Plasmodium* in the smear of the blood test, and Giemsa stained smears by parasitology labs from Kermanshah province. Patients were divided into three age groups: ≤5 years, between 5 and 15 years, >15 years. In addition, the nationality of the patients was divided into Iranian, Iraqi, and Afghan.

Data are expressed using descriptive statistics, including mean for quantitative variables and frequency percentage and two-dimensional agreement tables for qualitative variables. The significance of the relationship between some variables and malaria incidence was measured by proportional statistical tests. Statistical analysis was performed using the Chi-square test (df=1, p<0.05) was considered significant. The data was processed using SPSS16. The annual parasite incidence in the province in different years is equal to the ratio of the number of positive cases per year to the population from the province in the same year. This amount was calculated to ten thousand. Based on the division of the province into winter and summer points, the cities of Qasr Shirin, Sarpol-e Zahab, and Gilan-e-Gharb were classified as tropical, and the rest of the cities as cold. *Plasmodium* species were divided into *Vivax* and *Falciparum* (other species have not been seen in the province).

## RESULTS

### Malaria transmission in Kermanshah

In the last three decades, a total of 1545 positive cases of malaria were recorded in Kermanshah province. The distribution of these cases between the early and the last years of the research was non-symmetric. From 1986 to the beginning of 1996, 1374 cases (88.93%) of malaria were recorded, and the number of cases increased every year. During this time, the number of malaria cases in 100,000 people reached 12.25 from 0.55. Since 1986, the malaria control program changed, and malaria transmission started decreasing until reaching the "elimination" phase in 2010. Only a few cases or no cases have been reported since then. There were 171 (11.07%) positive malaria cases from 1986 to 2014. The incidence rate was tested with the Wilcoxon Signed Ranks Test, and the difference between the number of positive cases in 1986–1996 and 1997–2014 was shown.

### Effective factors on malaria transmission in Kermanshah province

The cities of Paveh, Javanroud, and Sarpol-e-Zahab had the most positive malaria cases in a population of 100,000 people. The rainfall amount in these cities was very similar to the malaria incidence, so we tested the correlation of rainfall and malaria incidence using the Spearman Rank Correlation test, which showed a positive and non-significant correlation. Consequently, we measured malaria incidence in the population, and the cities of Paveh, Javanroud, and Sarpol-e Zahab had the highest malaria incidence, respectively. The occurrence of malaria in each city was tested using the Spearman correlation, and a positive and significant relationship was seen. Sahneh, Songhar, and Harsin counties had the lowest number of cases in Kermanshah province in all studies (Table 1).

**Table 1.** Correlation between positive malaria cases (percent of cases and number of cases) in cities of Kermanshah province with annual rainfall

			Rain	Malaria %	Malaria number
**Spearman's rho**	**Rain**	**Correlation Coefficient**	1.000	0.436	0.436
		**Sig. (2-tailed)**	.	0.180	0.180
		**N**	11	11	11
	**Malaria %**	**Correlation Coefficient**	0.436	1.000	1.000**
		**Sig. (2-tailed)**	0.180	.	.
		**N**	11	11	11
	**Malaria number**	**Correlation Coefficient**	0.436	1.000**	1.000
		**Sig. (2-tailed)**	0.180	.	.
		**N**	11	11	11

Significance level: *.05, **.01, ***.001.

The average annual rainfall in the province and the annual malaria-positive cases in the province from 1986 to 2014 were tested with non-parametric tests. The Spearman Rank Correlation test showed a positive correlation. The correlation is significant at the 0.05 level (2-tailed) (Table 2).

**Table 2.** The average annual rainfall in the Kermanshah province and the annual malaria positive cases in the province from 1986 to 2014.

			Raining in Kermanshah	Malaria
**Spearman's rho**	**Raining in Kermanshah**	**Correlation Coefficient**	1.000	0.387*
		**Sig. (2-tailed)**	.	0.038
		**N**	29	29
	**Malaria**	**Correlation Coefficient**	0.387*	1.000
		**Sig. (2-tailed)**	0.038	.
		**N**	29	29

Significance level: *.05, **.01, ***.001.

Concerning the temperature, the number of positive malaria cases is three times higher in cold areas than in hot areas, but the prevalence (number of positive cases in 100,000 people) is three times higher in hot areas.

The age distribution shows two patterns in the early years (1986–1996) and the last years (1997–2014). In the first pattern, positive cases are primarily identified among people of young ages, but in the second pattern, the percentage of positive cases among young ages decreases. The average age is similar in both patterns (23.1 years). More positive cases were identified in people aged >15 years. This could be explained by the fact that this age group is outdoors after sunset (when the mosquito is active), and they could have more contact with the vector in this group.

The sex factor, similar to the age factor, has different patterns in two intervals. In the first interval, the prevalence of malaria-positive cases was higher among the female gender, which highly decreased in the second interval (32.5% to 17.3%). By 2000, no female positive case was reported. Overall, the male sex, with 68.32% of all positive cases, is the main infected gender. We know that gender is not a risk factor of malaria infection by itself. However, the higher probability of traveling to malaria foci, being exposed to insect bites outdoors, and migration for economic causes in males, makes them more susceptible to being infected. Regarding the *plasmodium* species, the majority was with *P. vivax* (98.38%) of all positive cases and *P. falciparum* (0.62%). No other species were ever seen in Kermanshah province.

Only 36 people of all 1545 positive malaria cases were non-Persian migrants. The majority were of Iraqi nationality (35 people), followed by Afghan nationality (1 person). No migrant cases have been seen since 1997. We performed the epidemiologic classification of transmission type in "imported" and "indigenous". In the first time interval, the indigenous transmission was the major way of transmission, and in the last interval, after local transmission cycle interruption, the transmission way shifted to "imported". There have been only 2 cases of indigenous transmission since 2002.

## DISCUSSION

During the study period, there was an increasing infection rate in the province. The incidence of malaria per 100,000 people increased by 97% from 0.55 in 1986 to 12.25 at the beginning of 1996. Since 1996, when anti-malaria policies underwent general changes, the process of reducing the incidence of malaria began, and by the end of 2014, a total of 171 cases (11.07% of the total) were reported. The incidence of the disease in these two time periods was measured by the Wilcoxon test, and there was a difference between the two periods. There is also a significant decrease in malaria incidence in the three southern provinces of Sistan & Baluchestan, Hormozgan, and Kerman. Following the implementation of the malaria control program in 1987, it seems that these three endemic cities have reached the "elimination" border [9]. In Khorasan Razavi province, positive malaria cases reached 494 cases in 2000 to 26 cases in 2008, decreasing by 94% [10]. The prevalence of malaria in Iran in 1988–1989 was 30–40 thousand cases annually, of which 80–85% was reported in the south and southeast region of the country [11]. The disease follows a similar pattern in other parts of Iran, *i.e.*, the north and center or the northern region of the Zagros and the western region. However, this is less evident in the southern and southeastern regions of the country [10]. The incidence of malaria in Kermanshah during these years has been less than 200 people per year, which is less than 1% of all malaria cases in the country.

On the other hand, it can be noted that from 1996 to 1999, about 143 cases of the disease were reported, of which 83.6% of all malaria cases were in the second period, and only 26 cases have been reported since 1993. Outbreaks appear to be exacerbated during the last four years of the first study period, 1992–1995 (as 83.7% of all cases). Therefore, an epidemic occurred during that time, starting in 1992 with an upward trend until 1994 and declining. However, after 1996, the decrease in malaria patients was more significant. Improving social and health status, together with the better and more coordinated implementation of national anti-malaria programs in the province and the use of Icon and Baygon insecticides instead of dichloro-diphenyl-trichloroethane (DDT), can be regarded as reasons for reducing malaria cases in the province. Since 2000, the incidence per 100,000 people dropped to less than 1, and since then, Kermanshah has reached the "elimination" stage. According to the WHO report on malaria statistics in Iran, there was a sharp increase to 90,000 cases per year in 1991 and a significant decrease to 12,294 cases in 2000 [8]. This sudden increase in Kermanshah and whole Iran, respectively, were the direct consequences of the economic and health problems caused by the Iran-Iraq war. However, in 2007–2011 in the city of Konarak in Sistan and Baluchestan province, malaria cases were 617, 329, 95, 79, 79, and 131 people, respectively, with an unexpected increase in 2007 compared to 2011. 1.2% of the study population has reached 2.07%, the sudden increases in temperature and hurricane Gonu Cyclone have created ponds and larval habitats, which increased the number of carriers [12].

From 2000 to 2013, malaria incidence in Iran decreased by more than 75% [10]. Between 2006 and 2014 alone, local malaria transmission went from 5,594 to 366, a 94% decrease over six years. During these 6 years, Kermanshah had 6 cases of malaria, all of which were of the incoming type. In 2012, 1629 cases of malaria were reported in Iran, and in 2013 it decreased by 50% compared to the previous year, to 519 cases per year [11]. This downward trend continued, and in 2014, 358 cases of malaria were reported in Iran [4]. Kermanshah did not have a local malaria transfer during these three years, and only one case of malaria from Zahedan was seen in Kermanshah in 2013.

In a study of Holakouee determining the epidemiological trend of malaria in Iran, 80 to 85% of malaria cases were in the south and southeast of the country in 1988, and 94.1% of total annual malaria in Iran in 1990 was in the three tropical provinces of Sistan and Baluchestan, Hormozgan, and Kerman [13].

The other study of Nazari, regarding the epidemiological indices of malaria in Kermanshah province (1996–2010), identified 168 malaria cases in Kermanshah province with about 2 million people. 94 (78.3%) of them were male, and in 163 cases (97%), *P. vivax* was the etiologic agent, and *P. falciparum* was found in 5 other cases. Most cases were reported in 1986, 87 patients (51.8%) and 43 (25.6%) cases were found in Paveh city [14].

In terms of age, people over the age of 15 make up the majority of people with malaria during the study years. Comparatively, between 1996 and the following years, the percentage of people over 15 years after 1996 significantly increased, and other age groups decreased. According to the sex ratio of patients in the province, as in all previous studies, males are more likely to be infected than females (68.32% *vs.* 31.67%). However, in both study periods, the number of female patients was lower than males (the first period was 67.5% for men and 82.7% for the second period). This difference in gender ratio as defined in the second period, *i.e.*, after eliminating local malaria transmission with the Kai-Esquire criterion for gender in two time periods, was significant (df=1 and p=0.004).

In the case of the *Plasmodium species,* the majority are with *P. vivax*, with 98.38% of the total cases. In other Iranian studies, *P. vivax* is the predominant species, for example, in the study of Mazandaran, the *P. vivax* accounted for 98.7% cases [15], and 91.5% cases in the study of Konarak [12]. In the study of Khorasan Razavi, only 30 of the 945 positive malaria cases in 7 years were *P. falciparum* type, and the majority of 96.4% was with *P. vivax* [10]. In our study, there were 25 cases of *P. falciparum* infection, 20 in the first period, and the other five between 1996–2000, 3 of which have traveled to the southeast regions. From 1979 onwards, there has been no infection with *P. falciparum* species in the province. This finding was also observed in the study of Hormozgan province, and during the years 81–86, the amount of *P. falciparum* decreased. In the study of Konarak, the cases of *P. falciparum* significantly declined from 31 cases in 2007 to 9 cases in 2011 [12].

Between 1986 and 2014 in the province, 36 people with malaria had non-Iranian nationalities, almost all Iraqi, and there was only one Afghan among them, contrary to other studies in other parts of the country. *P. falciparum* in Hormozgan province has been mainly imported and common for Afghan immigrants in this province. It is compatible with Kermanshah province in terms of imports, but Afghan immigrants in Kermanshah province do not have a share in the transfer of malaria.

In a study by Holakouee *et al.* on the epidemiological trend of malaria in Iran from 1941 to 2006, the highest rate of malaria in non-Iranians was 50% of the total cases in 2002, which has decreased since that year and reached less than 20% in 1985 [13]. At the same time, the epidemiological model of malaria in the country's southern regions has shifted from "imported" in 2002 and "local transfer", which is not in line with Kermanshah province. However, in the same study, in the early 1991s, the local transmission chain was cut off for the western part of the country, and the pattern shifted to "imported" from Iraq, Afghanistan, and other malaria-prone cities in Iran is consistent with our study.

## CONCLUSION

After the major changes in malaria control programs in Kermanshah province, the number of positive cases fell noticeably, and has reached the elimination stage. All malaria cases were "imported" in the last years, and no resistance type was ever seen.

## ACKNOWLEDGMENTS

### Conflict of interest

The authors declare no conflict of interest.

### Ethics approval

The protocol was approved by the Ethics Committee of Kermanshah University of Medical Sciences (KUMS.REC.1395.189).

### Funding

This study was supported by a grant from the Vice Chancellery for Research and Technology, Kermanshah University of Medical Sciences (grant number: 95170).

### Personal thanks

We appreciate the efforts and cooperation of the Parasitology Department of Kermanshah University of Medical Sciences and officials in the hygienic centers of Kermanshah province who played a significant role in advancing this research.

### Authorship

YH and NN developed the original idea, the protocol, and the study design. YH and PK collected and managed the data. MR participated in data analyses. YH and NN participated in drafting, and PK edited the manuscript. All authors provided comments, participated in writing the manuscript and approved the final manuscript.
